# Every hill has its leopard: patterns of space use by leopards (*Panthera pardus*) in a mixed use landscape in India

**DOI:** 10.7717/peerj.10072

**Published:** 2020-10-08

**Authors:** Sanjay Gubbi, Koustubh Sharma, Vijaya Kumara

**Affiliations:** 1 Nature Conservation Foundation, Mysore, Karnataka, India; 2 Department of Wildlife and Management, Kuvempu University, Shankaraghatta, Karnataka, India; 3 Holématthi Nature Foundation, Bengaluru, Karnataka, India; 4 Snow Leopard Trust, Seattle, WA, USA

**Keywords:** Distribution, Occupancy surveys, Human-dominated landscape, India, Large prey, Leopard, Natural habitats, Ecological variables, Space use, *Panthera pardus fusca*

## Abstract

Understanding abundance and distribution of species is often necessary for wildlife conservation. However, elusive species such as the leopard (*Panthera pardus*) that have wide geographical distribution and typically low abundance pose a constant challenge to conservationists due to logistical and methodological constraints. Although leopard abundance has been estimated at the scale of protected areas or other smaller regions, reliable information describing leopard distribution over large spatial scales remains largely unavailable. Knowledge about space use by leopards within landscapes could help improve conservation management, reduce human-wildlife conflict, and also facilitate population status monitoring. We carried out occupancy surveys across c. 24,000 km^2^ in southern India in a landscape that consisted a mosaic of leopards’ natural habitats and highly human-dominated areas. We investigated the effects of key ecological and anthropogenic variables in determining leopard space use patterns. We addressed imperfect detections obtained using sign surveys conducted on spatially replicated transects within sampling units by modeling detection as a function of spatial auto-correlation and covariates. Our results show that the probability of site-use by leopards across the landscape varied between 0.02 (95% CI [0.01–0.09]) and 0.99 (95% CI [0.99–1.0]) across the study area. The best model (AIC weight = 0.97) showed that the probability of leopard space use was affected by the proportion of natural habitats and the presence of large wild prey in the sampling unit. Given that India is undergoing rapid modifications due to economic changes and demand for natural resources, we emphasize the need for landscape-based approach for conserving and monitoring leopards. We argue that leopards are an indicator of functional ecosystems represented by scrub, deciduous forest and rocky outcrops that do not always get prioritized for conservation, unlike densely forested habitats. Similarly, conservation of natural large wild prey, especially outside the protected area system, should assume greater importance, which could also have a positive impact on reducing human-leopard conflict.

## Introduction

The leopard (*Panthera pardus*) is a habitat generalist with the widest distribution of all members of the genus Panthera (Stein & Hayssen, 2013). It is known to tolerate extreme variation in climatic conditions and habitat types where some subspecies survive in hot, desert and semi-desert regions, whereas others such as the Amur leopard (*Panthera pardus orientalis*) survive in sub-zero temperatures (Stein et al., 2020). This plasticity creates both opportunities and challenges for the conservation of the species. Some of the subspecies of leopards have been decimated from more than 70% of their historical range (Jacobson et al., 2016). Leopard populations are deteriorating due to habitat loss, poaching for body parts, loss of prey, vehicular collisions, retaliatory killing due to conflict, and other causes across its distribution range. They are also legally hunted in some countries (CITES, 2019). Most threats to leopards can be directly attributed to anthropogenic activities (Ray, Hunter & Zigouris, 2005; Henschel et al., 2011; Raza et al., 2012; Nowell, 2014; Durant et al., 2015; Stein et al., 2020; Jacobson et al., 2016; Gubbi et al., 2014, 2019). Due to the declining trends, the leopard was up-listed from the Near Threatened to the Vulnerable category by International Union for Conservation of Nature (2016).

Lack of data about distribution and abundance from sizable parts of its distribution range is considered to be an additional threat to leopard conservation (Stein et al., 2020). Even though a few studies on some of the subspecies report spatial distribution about leopards based on presence-only data (Al-Johany, 2007; Khorozyan et al., 2010; Laguardia et al., 2017), none of them address the uncertainty with false absences. In the absence of reliable information, much of the conservation decisions across large landscapes or geographical regions are based on expert opinions rather than sound scientific evidence.

Indices and abundance estimation have long been used to understand population distribution of species. Indices have limited value in monitoring programs, whereas abundance estimation is resource expensive and difficult to implement over large landscapes. Estimating probability of occupancy is widely used to determine and monitor the status of species, especially those which are known to be territorial and solitary (Wibisono et al., 2011; Santika et al., 2014; Carter et al., 2015). Presence-absence surveys often rely on evidence of the presence of a species in a particular area of interest, but seldom capture absence with any level of accuracy. One of the biggest challenges elusive predators such as leopards pose to researchers is that of imperfect detection (Linkie et al., 2007, 2010; Wibisono et al., 2011; Taubmann et al., 2016). Unless used in a framework that corrects for detection probability that is often less than one, and addresses its likely dependance on several confounding factors, reports based on presence-only data are likely to be biased and mostly unreliable.

Recent developments in empirical estimation of probability of occupancy (ψ) that incorporates imperfect probability of detection (*p*) provide researchers with a powerful tool to estimate species distribution (MacKenzie et al., 2003, 2006). By incorporating estimates of detectability, this approach corrects the inherent negative bias present in naïve occupancy estimates (MacKenzie et al., 2003), thus making occupancy-based models useful in studying elusive species (Linkie et al., 2007; Wibisono et al., 2011; Sunarto et al., 2012; Ghoshal et al., 2019; Taubmann et al., 2016). Occupancy models have also been used to estimate leopard populations, habitat use, activity rates, corridor functionality, livestock-leopard interactions and other ecological aspects (Ghoddousi et al., 2010; Carter et al., 2015; Sasidharan et al., 2016; Thapa et al., 2017; Pudyatmoko, 2017). In this study, we estimate the probability of leopard occupancy as a function of natural, anthropogenic and geographic covariates, and estimate the probability of detecting leopards using indirect signs as a function of spatial and observation-specific covariates.

Leopards use a wide range of heterogeneous habitats including highly human-dominated areas, forests, rocky outcrops and savannah. Our goal was to understand the spatial distribution of leopards in a largely human-dominated landscape, and to assess ecological and anthropogenic variables that impacted space use by leopards in the study area. This would help provide insights for conservation planning and appropriate management interventions to reduce human-leopard conflict.

Habitats outside Protected Areas (PAs) can support relatively high densities of leopards (Gubbi et al., 2017a) and act as important corridors or stepping stones (Chundawat, 2018) that maintain landscape integrity and therefore population viability. Maintaining connectivity through such habitats have shown to benefit large carnivores, including leopards, to maintain genetic variability, avoid genetic bottlenecks, acting as sink sites to maintain landscape-level population viability both in India and elsewhere even when such habitats contain high human densities (Dutta et al., 2013a, 2016; Sharma et al., 2013; Carter et al., 2015; Gubbi et al., 2017b; Farhadiniaa et al., 2015; Ashrafzadeh et al., 2020).

In India, space-use by leopards, especially outside PAs has been rarely studied other than some recent assessments of their populations in agricultural landscapes (Athreya et al., 2013). In contrast, most studies have largely focused on population estimation within PAs (Harihar, Pandav & Goyal, 2009; Kalle et al., 2011; Borah et al., 2014). However, leopards range beyond PAs into multiple-use forests and even in agricultural landscapes. Unfortunately, conservation programs have tended to overlook leopard habitats outside the PA system which are under immense pressure from natural resource extraction, habitat loss and degradation, and severe loss of natural prey.

India has a human population of over 1.3 billion and an economy poised at a growth rate of 7% annually. This growth comes with the development of associated infrastructure, urbanization and expansion of agriculture, many of which could fragment wildlife habitats, especially for large mammals. In this background, it is critical to secure wildlife landscape structures that ensure functional connectivity, which can then facilitate dispersal and long-term persistence of wildlife. With tigers, Thatte et al. (2018) illustrate that the local extinction probability could decline by 68% if habitat corridors were established. Studies from the Central Indian landscape demonstrate that leopards were able to maintain genetic exchange and diversity due to the existence of functional corridors between PAs (Dutta et al., 2013b; Thatte et al., 2018). For leopards, built-up area, human population and roads are known to affect their genetic flow (Thatte et al., 2020). India’s PA network forms only 5% of its geographical area. Therefore, ensuring connectivity between PAs, and habitats outside PAs can play a critical role in maintaining metapopulation structure for large carnivores. Understanding space use by leopards outside PAs is thus important to identify patches that are used by leopards and to identify correlates that can help in effective conservation planning.

Considering the severe lack of data about how leopards use space, there is an urgent need to (1) obtain reliable estimates of the probability of leopard space use across a multi-user landscape; and (2) investigate the effect of key ecological and anthropogenic variables correlating with space use patterns of leopards. In this study, we estimate the probability of space use by leopards in a multi-user landscape represented by a mosaic of natural habitats and areas with high human presence in the vicinity of the megapolis, Bengaluru. As an elusive species, leopards can often go undetected despite presence. We use occupancy (ψ) framework that incorporates empirical estimation of detection probability (*p*) to estimate the probability of space use by a species in an area of interest (MacKenzie et al., 2006). Our results provide valuable baselines that can be used to assess the role of ecological and anthropogenic factors on leopard habitat use, with a methodology that can be applied to monitor shifts in the spatial distribution of leopards in India.

## Materials and Methods

### Study area

**Figure 1:**
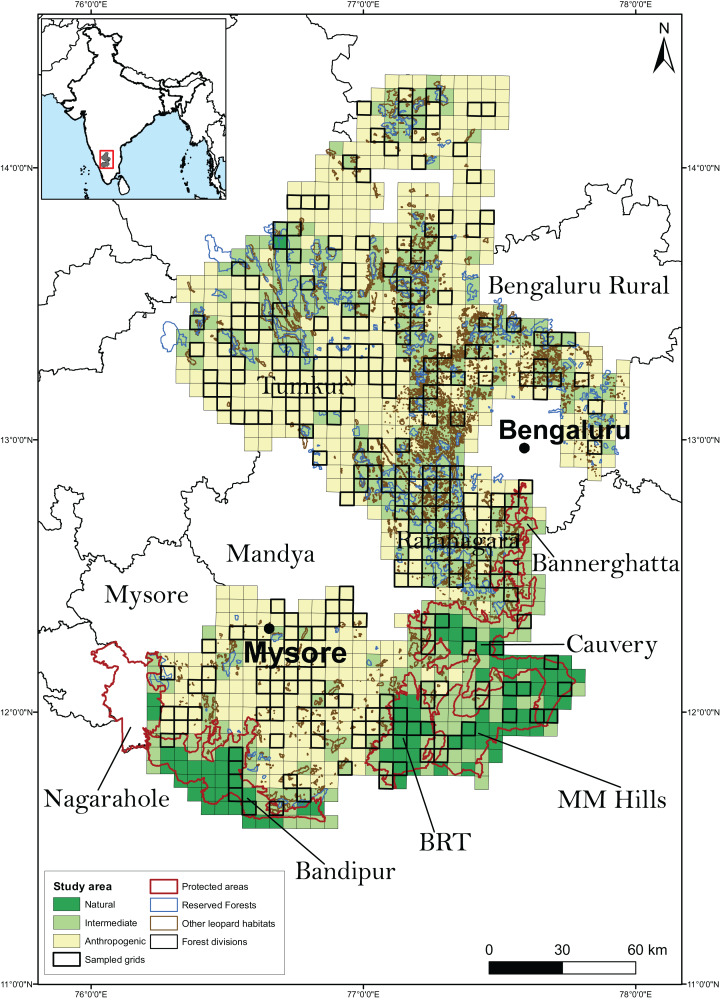
Study area along with the sampled grids. The study area, broadly classified as on the basis of predominant land use type, was divided into 1,058 grid-cells (sampling units) of 30 km^2^ each. A total of 267 sampling units were randomly chosen to be surveyed for leopard presence.

Research permissions for our field surveys were provided by the Karnataka Forest Department (Approval no: C1/WL/CR-2011-12 and PCCF/C/GL-35/2012-13). The study was conducted in the southern Indian state of Karnataka (Fig. 1). The study landscape (23,902 km^2^) encompassed 3,601 km^2^ of PAs (Bannerghatta National Park, Bandipur and BRT Tiger Reserves, Cauvery and MM Hills Wildlife Sanctuaries), 1,399 km^2^ of multiple-use forests (reserved and state forests), 1,183 km^2^ of leopard habitats such as rocky outcrops that are not protected under any conservation laws and 19,719 km^2^ of highly human-settled landscapes such as agricultural fields and fallow land. Leopards are generalists in habitat use and are known to use a diversity of habitats including scrub, dry, moist deciduous, semi-evergreen, evergreen forests, woodland savanna and even agricultural fields (Balme, Hunter & Slotow, 2007; Simcharoen et al., 2008; Athreya et al., 2013). These areas support a diverse assemblage of wild ungulates and other principal leopard prey species including gaur (*Bos gaurus*), sambar (*Rusa unicolor*), chital (*Axis axis)*, barking deer (*Muntiacus vaginalis*), four-horned antelope (*Tetracerus quadricornis*), wild pig (*Sus scrofa*) and others. It also hosts domestic livestock and dogs that act as leopard prey. Conspecific large predators in the study area included tigers (*Panthera tigris*), dholes (*Cuon alpinus*) and striped hyena (*Hyaena hyena*).

The PAs predominantly consists of dry deciduous, moist deciduous and woodland savannah forests. However, some parts of BRT Tiger Reserve also consist of evergreen and high elevation “shola” forests. Such habitats are also found in small areas of Bandipur Tiger Reserve and MM Hills Wildlife Sanctuary. The multiple-use forests are primarily dry deciduous and scrub forests. The highly human-settled areas include a mosaic of agricultural fields, human settlements, plantations (eucalyptus, areca nut, coconut, sugarcane and others), fallow lands and water bodies (lakes, tanks and reservoirs).

The human population in the study area is estimated to be >8.7 million with a density of 329 people/km^2^ (Directorate of Census Operations, 2011). For detailed accounts of the study area, see Gubbi et al. (2017a). The study area is characterized by anthropogenic pressures such as poaching of leopard prey, quarrying, mining and urbanization that potentially impact leopard distribution and abundance. Our sampling design represented all available leopard habitat types in the study area.

### Survey design

We used the freely available land use land cover imagery from the year 2011–2012 of 1:50,000 resolution (http://bhuvan.nrsc.gov.in/gis/thematic/index.php) to classify various land use categories (irrigated or unirrigated agricultural habitats, built-up area, natural forests, fallow land and water bodies). We used the geographical information system (GIS) layers available from the Karnataka State Remote Sensing Applications Centre for calculating the area under reserved forests and PAs. Finally, other natural leopard habitats such as rocky outcrops were digitized using Google Earth imagery (Google LLC 7.1, 2013). Nearly 72.5% of the digitized habitats were ground-truthed in January and February 2014 to verify their status by visiting the location and visually scanning for habitat type.

Based on the availability of the above information, we divided the study area into 1,058 grid cells (sampling units) of size 30 km^2^ each. We chose the sampling unit size to be comparable to the known home range of leopards reported from earlier studies (Norton & Henley, 1987; Grassman, 1999; Odden & Wegge, 2005; Athreya et al., 2010; Odden et al., 2014), even though we did not expect spatial closure as occupancy was interpreted as the probability of site use (Mackenzie & Royle, 2005). Moreover, since we did not intend to equate occupancy with abundance, and that there were no discernible features on the landscape that would serve as boundaries for individual animals, we conceded that there was no considerable advantage in defining sampling units larger than the leopard’s home range (MacKenzie et al., 2018). The sampling units were also categorized as predominantly natural (units with >90% leopard natural habitat), intermediate (units in-between) and predominantly anthropogenic (units with <10% leopard natural habitat). A total of 267 (25%) sampling units were randomly selected to conduct the sign surveys for estimating leopard occupancy (Fig. 1). The adequacy of the sampling was investigated by plotting the distribution of sampled units along the breadth of the relevant covariates from the best models (see “Methods and Results” section).

### Field surveys

We carried out sign surveys within 267 randomly selected sampling units. Prior to the surveys, pilot training surveys were carried out to familiarize the researchers with field protocols in a subset of sampling units. We conducted surveys to determine leopard presence through indirect signs such as pugmarks and feces. Leopards use trails and dirt roads extensively for patrolling their territory and/or hunting, and it is reasonable to expect them to show up on roads or trails if they are using the space represented by the 30 km^2^ sampling unit. Our survey teams consisting of 2–3 skilled researchers in mammal sign recognition walked on trails and dirt roads to identify, record and geo-reference leopard signs (tracks, feces and direct sightings). We spent adequate time to verify the signs and only fresh and correctly identifiable signs were recorded. Each sign was photographed and cross verified with senior researchers to avoid any misclassifications.

If the sign could not be recorded with absolute certainty it was omitted to avoid false detections. We also recorded any evidence of the presence of large and small prey species through direct sightings and indirect signs. Apart from the animal signs, we also recorded data on the evidence of presence of threats such as hunting, quarrying and mining that could have either affected leopard space use or influenced our ability to detect its presence. We sampled 10 km long transects, making a conscious effort that natural habitats and unirrigated and irrigated agricultural habitats were surveyed in proportion to their extent within each sampled unit. The 10 km trails were broken into 1 km segments to be treated as spatial replicates. Detections or non-detections of evidence of leopard presence within each 1 km segment were recorded as “1” and “0” respectively.

To minimize the effect of climatic factors such as rain, field surveys were carried out during the dry seasons between March 2014 and June 2014, and September 2014 and November 2014. Our teams traversed 2,768 km in transects, yielding a total of 240 detections of leopard.

### Covariates for leopard occupancy

We hypothesized that leopards depend on the proportion of natural habitats as opposed to irrigated and unirrigated agricultural habitats. We also predicted that presence of large (>20 kg: gaur, sambar, chital, wild boar, four-horned antelope and barking deer) and small-bodied (<20 kg: bonnet macaque (*Macaca radiata*), gray langur (*Semnopithecus priam*), Indian hare (*Lepus nigricollis*), Indian crested porcupine (*Hystrix indica*), Indian peafowl (*Pavo cristatus*) and gray junglefowl (*Gallus sonneratii*)) wild prey species played an important role in the distribution of leopards even in the human-dominated landscape. Lastly, we expected areas with mining to have a negative effect on the distribution of leopards. Since we used animal trails to detect leopard presence, we expected detections to be spatially correlated. We anticipated the presence of livestock, dogs, and mining operations to affect our teams’ ability to detect leopard tracks due to disturbance and destruction of signs. We also investigated if detection of leopards was affected by micro-habitat categories such as proportions of sampled patches represented by agricultural land, dry deciduous forest, eucalyptus plantation, grassland, moist deciduous forest, orchards, scrub forest or rocky terrain. We expected detection of leopard signs to be higher in natural habitats as opposed to unirrigated and irrigated agricultural patches due to less human movement in the former. Since all surveys were done during similar weather conditions by teams that were mixed up randomly, we did not test the effects of survey teams on detection probability.

To develop site covariates, GIS layers of all sampling units were uploaded on Google Earth and visually scanned for the presence of quarries and mines within the sampling units. Time Series imagery in Google Earth was also used to verify the change in land-use from natural habitats that were converted as quarries/mines within the units. Time series imagery also helped clarify any ambiguities as some of the locations that seemed as quarries were dry lakebeds or other developmental activities. Elevation profile also helped to distinguish between quarries and mines by visualizing the positive or negative slope. The government’s online satellite imagery Bhuvan (www.bhuvan.nrsc.gov.in) was used to cross-verify images on Google Earth. All identified quarries and mines were digitized and the polygons were used to calculate the area of the quarry/mine across the study area. Further, ground-truthing was carried out amongst randomly selected quarries/mines to ensure that the digitized data complied with ground information. Presence of quarries and mines were recorded as a binary covariate. Natural habitats, and unirrigated and irrigated agricultural habitats were classified using satellite imagery and proportion of each habitat category within the sampling unit was estimated and used as contiguous site-covariates. A categorical covariate was also created to investigate the space use by leopards as a function of whether the sampling units were predominantly natural, intermediate or predominantly anthropogenic. We assumed nearly perfect detection of large and small-bodied prey since it was unlikely for our teams to miss each one of the six large-bodied and six small-bodied prey species from the sampling units, given presence. Presence or absence of any one of the large-bodied and small-bodied prey was determined by extensive searches within each sampling unit and used in the analysis as binary covariates. All covariates were transformed so as to have a mean of “0” and a standard deviation of “1” before analysis.

### Statistical analyses and modeling leopard occupancy

The surveys were conducted using line transects with 1km segments considered as survey replicates (Kendall & White, 2009). We used the package rPresence v 2.0 (Proteus, Dunedin, New Zealand) to run our analysis in R programing interface (RStudio, Inc. 2009–2013). Effects of various covariates on probability of site use by leopards (ψ) and the probability of detection (*p*) were investigated through information-theoretic approach that uses AIC to choose the best models describing the dataset (Burnham & Anderson, 2002). We developed an ecologically meaningful candidate model set with 58 models investigating the effect of covariates on probabilities of occupancy and detection. We also estimated c-hat from the most parameterized model to test the goodness of fit. For a model with an adequate description of data, c-hat is expected to be close to “1”, whereas greater and smaller values represent overdispersion and underdispersion respectively (Burnham & Anderson, 2002). Since we anticipated spatial correlation in detecting leopards on trails, we ran the models that correct for correlated detections (Hines et al., 2010). These models use Markovian dependance of animal detection on spatial replicates and also account for spatial auto-correlation of detection on contiguous replicates (θ^1^_i_ defined as the probability of leopard being available for detection in the *i*th segment conditional to its presence or absence in the (*i* – 1)th segment). We compared these models with those that assumed no spatial correlation in detection, and also investigated the effects of covariates discussed above.

## Results

We surveyed 267 sampling units, covering 23,902 km^2^ of leopard habitat within our study landscape. Within each unit, we surveyed several 1 km long spatial replicates (mean effort per sampling unit = 10 km) resulting in a total walk effort of 2,705 km. We detected leopards on 86 out of the 267 sampled units, producing a naive occupancy estimate of 0.32. We developed and ran 58 models using an ecologically meaningful candidate set. We made sure not to use covariate combinations that had a high correlation coefficient (*r* > 0.7). The c-hat estimate of 1.05, close enough to 1.0 indicates that the most parameterized model was an adequate description of the data (Mackenzie & Bailey, 2004).

**Figure 2:**
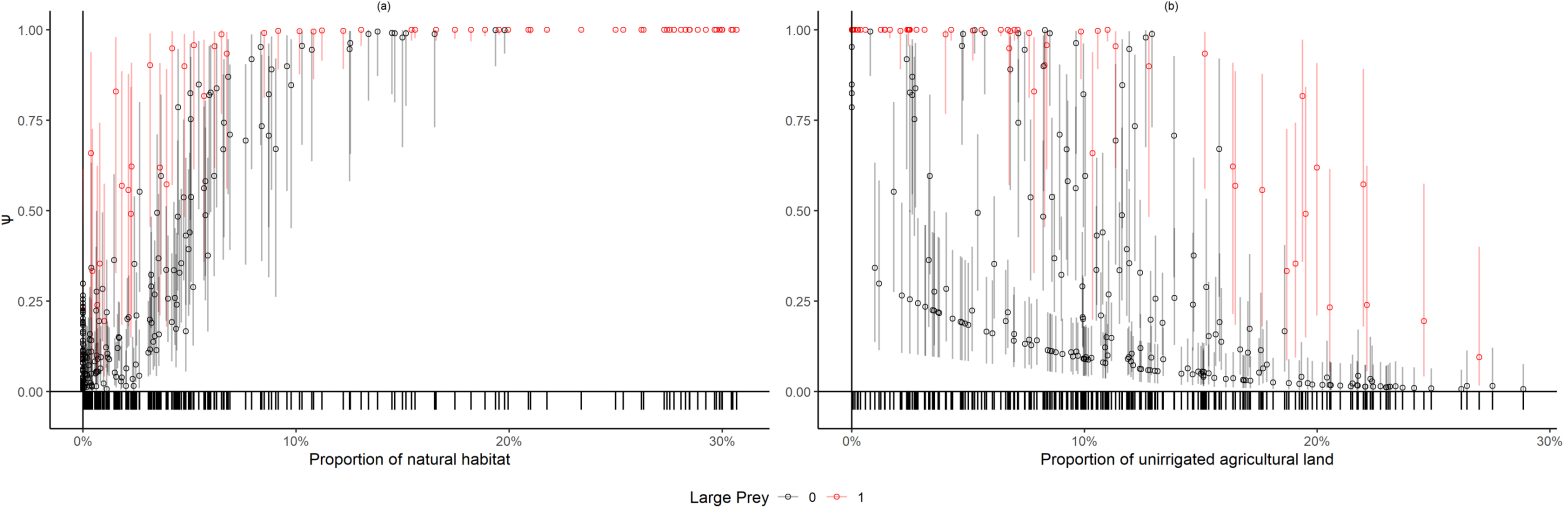
Variation in leopard occupancy (ψ) across the sampled 267 units. Variation in leopard occupancy (Ψ) across the sampled 267 units and the corresponding 95% confidence intervals using the top model chosen by AIC, plotted against proportion of (A) natural and (B) unirrigated agricultural habitat in the sampling units. Red and black points denote sampling units with and without large prey presence respectively. The tick-marks on the *x*-axis denote locations of sampling units on the covariate space.

We present the AIC table listing top 10 models (Table 1) even though the cumulative weight of the first four models summed up to 1.0. The covariates used in the top models indicate that extent of natural habitat was the most important covariate explaining the probability of leopard space use (Ψ), followed by the extent of unirrigated agricultural area within sampling units. The model coefficients from the top models (Table 2) indicate that probability of sites being used by leopards increased with the extent of natural habitat on the logit scale (β_natural_= 4.00, SE = 1.13, Fig. 2A) and was greater for sampling units with large prey (β_large prey_ = 2.85, SE = 1.13, Figs. 2A and 2B). Probability of site use reduced for an increase in the proportion of unirrigated agricultural habitat (β_unirrigated_ = −1.18, SE = 0.44, Fig. 2B). Our models also indicated that leopard detections on trails were likely to be spatially correlated (ϴ^0^ = −2.21, S.E.= 0.22; ϴ^1^= 3.31, S.E. = 0.38, where ϴ^0^ denotes the probability that a species is available in a transect segment given that it was not available in the previous segment; and ϴ^1^ denoting the probability that a species is available in a transect segment given that it was available in the previous segment). Additionally, coefficients of covariates for detection probability suggested that leopards were better detected on transect segments that were on grasslands (β_grassland_ = 0.21, SE = 0.30). The average occupancy for the sampled units based on the top model was estimated to be 0.43 (SE = 0.4), though it ranged between 0.02 (95% CI [0.01–0.09]) and 0.99 (95% CI [0.99–1.0]).

**Table 1:** The different models ranked on the basis of AIC.

Model No.	Model format	ΔAIC	AIC weight	npar	Likelihood
1	psi (~Natural + Unirrigated + LargePrey), theta(~PRIME), p(~Grassland)	0	0.9757	8	1120.93
2	psi(~Natural + Unirrigated), theta(~PRIME), p(~Grassland)	7.99	0.018	7	1130.92
3	psi(~Natural), theta(~PRIME), p(~Grassland)	11.34	0.0034	6	1136.27
4	psi(~Natural), theta(~PRIME), p(~1)	11.59	0.003	5	1138.53
5	psi(~Category), theta(~PRIME), p(~Grassland)	34.74	0	7	1157.67
6	psi(~Unirrigated), theta(~PRIME), p(~Grassland)	58.75	0	6	1183.69
7	psi(~LargePrey), theta(~PRIME), p(~Grassland)	62.68	0	6	1187.61
8	psi(Natural + Unirrigated + LargePrey), p(MoistDec + Grassland + IrrigatedSeg)	94.48	0	8	1217.41
9	psi(Natural + LargePrey + irrigated), p(MoistDec + Grassland + IrrigatedSeg)	95.19	0	8	1218.12
10	psi(Natural + Unirrigated + LargePrey + Irrigated), p(MoistDec + Grassland + IrrigatedSeg)	96.3	0	9	1217.23

**Notes:**

Top 10 models from the candidate model set of 58 models, ranked on the basis of minimum AIC. All site covariates are averaged for the entire sampling grid (5 × 5 km), and the sampling covariates are estimated for each transect segment (1 km).

Parameters: Δ*AIC*, difference between the AIC and the minimum AIC for the given candidate model set; *AIC weight*, relative likelihood of the model; *npar*, number of parameters used in the model; *Likelihood*, likelihood of the model; *psi*, probability of a sampling unit being used by a leopard; *theta(~PRIME)*, probability of leopard being available for detection in a segment was dependent on their availability (or unavailability) in the preceding segment; and *p*, detection probability of leopard.

**Table 2:** Top models and the relative importance and effects.

Estimate	Covariate	Cumulative AIC weight	Coefficient (±SE)		
			Model 1	Model 2	Model 3
			0.98	0.018	0.003
Occupancy	Intercept	1	0.44 ± 0.61	0.65 ± 0.61	0.8 ± 0.63
	Natural	1	4.00 ± 1.13	3.82 ± 1.10	4.32 ± 1.15
	Unirrigated	0.99	−1.78 ± 0.44	−0.69 ± 0.3	–
	Large Prey	0.98	2.85 ± 1.13	–	–
Conditional detection probability	Theta0	1	−2.21 ± 0.22	−2.16 ± 0.22	−2.22 ± 0.22
	Theta1	1	3.31 ± 0.38	3.23 ± 0.39	3.31 ± 0.38
Detection probability	Intercept	1	0.47 ± 0.23	0.48 ± 0.23	0.48 ± 0.23
	Grassland	1	0.21 ± 0.30	0.21 ± 0.32	0.21 ± 0.31

**Note:**

Top models, their AIC weights and coefficients of covariates depicting their relative importance and effects.

**Figure 3:**
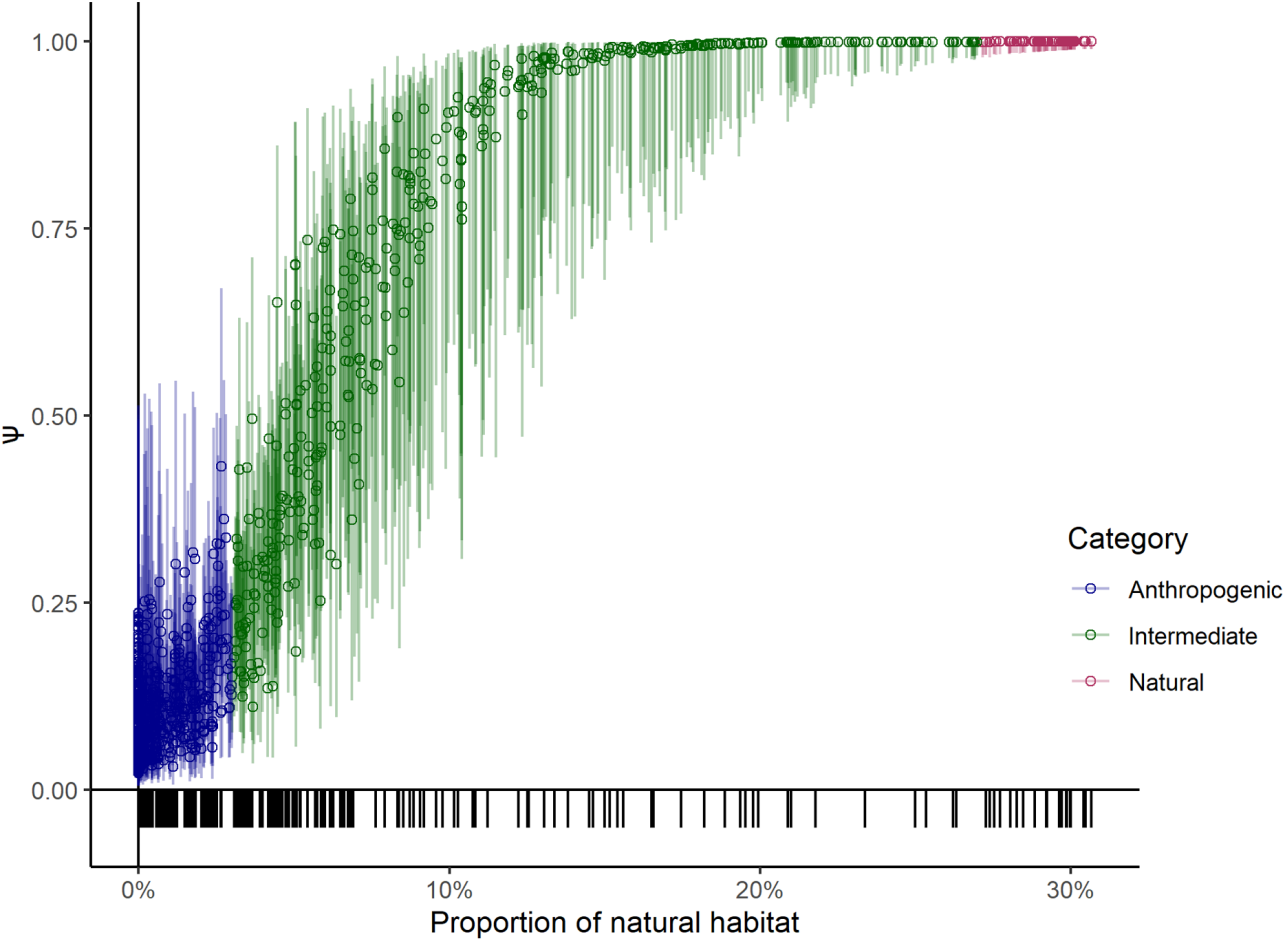
Probability of leopard occupancy (Ψ) and its corresponding 95% confidence intervals predicted for the entire study area. Probability of leopard occupancy (Ψ) and its corresponding 95% confidence intervals predicted for the entire study area using the second best model, plotted against transformed proportion of natural habitat in the sampling units. The tick-marks on the *x*-axis denote locations of sampling units on the entire covariate space.

**Figure 4:**
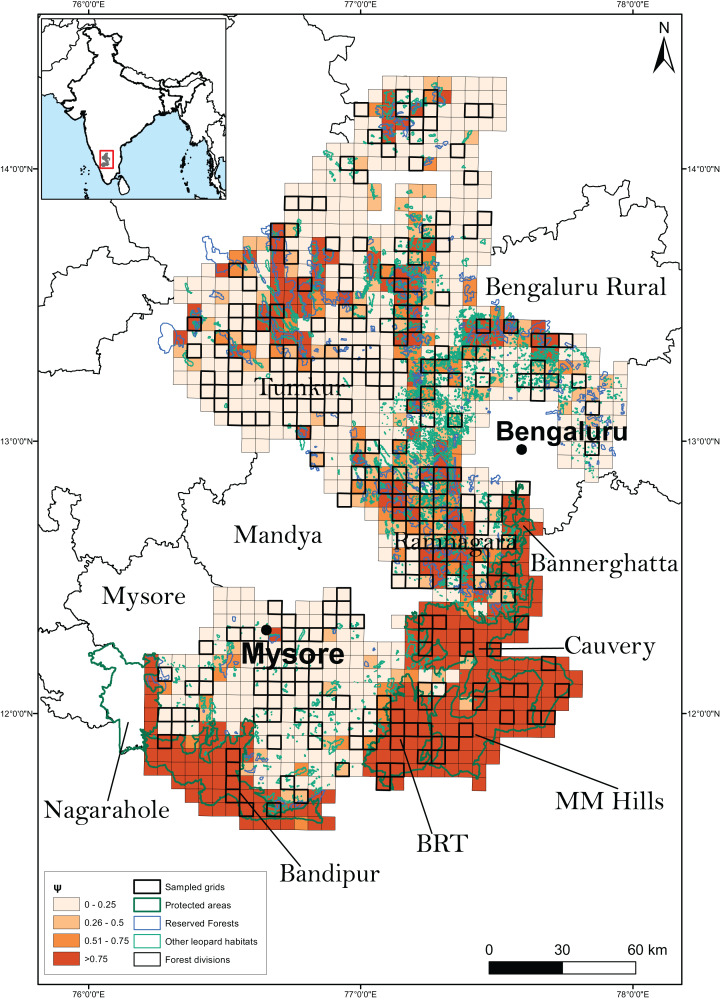
Results of the second best model to predict leopard occupancy. Probability of site use by leopard predicted across the 23,902 km^2^ of the entire study area using the second best model assuming availability of large bodied wild prey to be constant throughout the landscape.

One of the covariates (large wild prey presence) in the top model was only available for the sampled units, therefore we could not use it to project leopard occupancy from the entire study area of 23,902 km^2^. Instead, we compared the coefficients of covariates of the top two models that only differed in using large wild prey presence as one of the site covariates to explain the variation in data and found that the coefficients of the covariates (Table 2) were similar. Our sampling effort was also representative of the entire covariate breadth representing the whole study area (Fig. 3). We thus used the results from the second-best model to predict leopard occupancy from the entire study area, that represents a scenario where the presence of large prey is assumed to be constant (Fig. 4).

## Discussion

### Overall occupancy and effects of covariates on detectability and occupancy

This is the first study of its kind that systematically investigates leopard occupancy across a large landscape characterized by a spectrum of diverse forested and human-dominated habitats. The predicted probability of site-use by leopards across the study area provides a baseline against which changes in the distribution of leopards can be assessed in the future. Our results show that leopard space use is overwhelmingly influenced by the proportion of natural habitat and presence of large wild prey within sampling units and that they are still vital for the survival of leopards, an adaptable and vagile species. Our findings corroborate other studies that highlight the importance of natural habitats and wild prey availability for leopards (Khorozyan, 2003; Khorozyan, Malkhasyan & Abramov, 2008; Wang et al., 2017; Abade et al., 2018; Strampelli et al., 2018).

The probability of leopard space use was positively associated with the proportion of natural habitat in the sampling unit and declined with an increase in the area of unirrigated agricultural lands (Fig. 3). The lower estimated probability of occupancy in highly human-dominated areas adds valuable information to another study on leopards in agricultural landscapes (Athreya et al., 2013). We suspect that even though leopards use areas with high human use, natural habitats provide them, especially sub-adults, with suitable habitat patches for dispersal and non-dispersal movements (Fattebert et al., 2015).

Although leopard occupancy was documented in human-settled areas it was likely occurring at the cost of livestock losses which is one of the primary causes of human-leopard conflict (Gubbi, Kolekar & Kumara, in press). Our results indicate that native wild prey plays a critical role in leopard conservation as leopards tend to shift to livestock depredation if wild prey becomes scarce (Shehzad et al., 2015).

### Conservation implications

Our findings clearly show that conservation of natural habitats and protection against loss of large wild prey such as sambar, chital, four-horned antelope is critical for leopards even within habitats outside PAs. Even though large wild prey did occur outside of PAs, their prevalence was extremely low which could be due to high levels of poaching outside PAs (S. Gubbi, 2018, unpublished data). Naha, Sathyakumar & Rawat (2018) argue that livestock potentially draws leopards to human-settled areas leading to conflict and such visitations augment the chance of human-wildlife interface at times leading to injuries or loss of human life. Therefore, efforts towards the protection of large wild prey outside PAs could also help reduce human-leopard conflict.

Though conflict is indeed a predictable outcome of overlap between leopards and people/livestock, the potential ecological drivers of conflict remain poorly understood. Therefore, to conserve leopards outside PAs, our study serves as the natural starting point for further research that may help predict where conflicts are likely to take place.

Given that our study landscape supports >8.7 million people and is undergoing rapid modifications due to economic changes and demand for natural resources, there is a need to prioritize areas for leopard conservation and economic development. In India, many areas where leopards are found also hold rich mineral deposits and are mined/quarried extensively. In our study site, 136 (50.9%) of the 267 surveyed sampling units had the presence of quarries or mines with a mean area of 1.44 ha/grid (0.012–33.898). Although the influence of mineral resource extraction on leopard space use was not evident in our model selection procedure, such activities pose a pervasive threat to remaining patches of natural leopard habitats in the unprotected areas of our study region. Therefore, identifying a network of patches with natural habitats (reserved forests and rocky outcrops) as non-mining/quarrying zones does not only provide ideal resting and denning refuges for a highly mobile species such as the leopard, but also minimize human-wildlife conflict. We emphasize that for leopard conservation, a landscape approach is imperative, where the leopard can be recognized as an umbrella species for functional ecosystems represented by scrub, deciduous forests and rocky outcrops that do not always classify as densely forested habitats.

## Conclusions

Even though abundance and densities are critical parameters for population monitoring to help management, occupancy surveys could be valuable for assessing species distributions and monitoring changes across large spatial scales. This method could be particularly useful for a species such as the leopard that has a wide distribution range. Estimating leopard abundance and density in habitat patches using spatial capture-recapture methods can be prioritized for specific habitat types, PAs or Reserved Forests depending on the need, capacity and resource availability (Williams, Nichols & Conroy, 2002; Witmer, 2005). However, for scales that extend to several thousand square kilometers of mixed-use landscapes, monitoring space use and changes in the form of local extinction and local colonization using occupancy-based models could serve as a reasonable surrogate for monitoring biodiversity.

The leopard is currently widespread and not severely threatened in India, even though it runs the risk of rapid declines in abundance and local extinctions in response to economic development and land-use changes, especially outside PAs. Although some of the leopard subspecies are sharply declining (Jacobson et al., 2016), India continues to be the stronghold of P.p. *fusca*. We find leopards occupying habitat patches outside PAs, but also suspect that unnatural mortality of leopards outside PAs could be much higher (Gubbi et al., 2014, 2019) than in PAs. It is also likely that the illegal trade in leopard body parts (Raza et al., 2012; Mondol et al., 2015) is thriving with leopards poached and supplied from outside PAs. The need to prioritize conservation action and monitoring of leopards within and outside PAs is thus an important issue not only to reduce human-leopard conflict but also to combat illegal wildlife trade.

## Supplemental Information

10.7717/peerj.10072/supp-1 Supplemental Information 1 R script code used for running the analysis.

10.7717/peerj.10072/supp-2 Supplemental Information 2 Raw data of occupancy sign surveys of leopards, its prey and other covariates.

10.7717/peerj.10072/supp-3 Supplemental Information 3 Detection of leopards during occupancy surveys.

10.7717/peerj.10072/supp-4 Supplemental Information 4 R script for leopard occupancy.

10.7717/peerj.10072/supp-5 Supplemental Information 5 Data on sampling covariates.

10.7717/peerj.10072/supp-6 Supplemental Information 6 R script used for different models.

10.7717/peerj.10072/supp-7 Supplemental Information 7 Data on site covariates.

## Additional Information and Declarations

### Field Study Permissions

The following information was supplied relating to field study approvals (i.e., approving body and any reference numbers): The field surveys were carried out with permissions from the Karnataka Forest Department (C1/WL/CR-2011-12 and PCCF/C/GL-35/2012-13).

### Data Availability

The following information was supplied regarding data availability: The R script code and data used for running the occupancy analysis are available in the Supplemental Files.

## References

[ref-71] Abade L, Cusack J, Moll RJ, Strampelli P, Dickman AJ, Macdonald DW, Montgomery RA (2018). Spatial variation in leopard (*Panthera pardus*) site use across a gradient of anthropogenic pressure in Tanzania’s Ruaha landscape. PLOS ONE.

[ref-1] Al-Johany AMH (2007). Distribution and conservation of the Arabian Leopard *Panthera pardus nimr* in Saudi Arabia. Journal of Arid Environments.

[ref-2] Ashrafzadeh MR, Khosravi R, Adibi MA, Taktehrani A, Wan HY, Cushman SA (2020). A multi-scale, multi-species approach for assessing effectiveness of habitat and connectivity conservation for endangered felids. Biological Conservation.

[ref-3] Athreya V, Odden M, Linnell JDC, Karanth KU (2010). Translocation as a tool for mitigating conflict with leopards in human-dominated landscapes of India. Conservation Biology.

[ref-4] Athreya V, Odden M, Linnell JD, Krishnaswamy J, Karanth U (2013). Big cats in our backyards: persistence of large carnivores in a human dominated landscape in India. PLOS ONE.

[ref-5] Balme G, Hunter L, Slotow R (2007). Feeding habitat selection by hunting leopards *Panthera pardus* in a woodland savanna: prey catchability versus abundance. Animal Behaviour.

[ref-6] Borah J, Sharma T, Das D, Rabha N, Kakati N, Basumatary A, Ahmed MF, Vattakaven J (2014). Abundance and density estimates for common leopard *Panthera pardus* and clouded leopard *Neofelis nebulosa* in Manas National Park, Assam, India. Oryx.

[ref-7] Burnham KP, Anderson DR (2002). Model selection and multimodel inference: a practical information-theoretic approach.

[ref-8] Carter N, Jasny M, Gurung B, Liu J (2015). Impacts of people and tigers on leopard spatiotemporal activity patterns in a global biodiversity hotspot. Global Ecology and Conservation.

[ref-9] Chundawat RS (2018). The rise and fall of the emerald tigers: ten years of research in Panna National Park.

[ref-10] CITES (2019). CITES national export quotas. https://citesorg/eng/resources/quotas/export_quotas?field_party_quotas_tid=&field_full_name_tid=&field_export_quotas_year_value%5Bvalue%5D%5Byear%5D=2018&items_per_page=50.

[ref-11] Directorate of Census Operations (2011). Primary census abstract data highlights Karnataka Series 30.

[ref-12] Durant SM, Becker MS, Creel S, Bashir S, Dickman AJ, Beudels-Jamar RC, Lichtenfeld L, Hilborn R, Wall J, Wittemyer G, Badamjav L, Blake S, Boitani L, Breitenmoser C, Broekhuis F, Christianson D, Cozzi G, Davenport TRB, Deutsch J, Devillers P, Dollar L, Dolrenry S, Douglas-Hamilton I, Dröge E, FitzHerbert E, Foley C, Hazzah L, Hopcraft JGC, Ikanda D, Jacobson A, Joubert D, Kelly MJ, Milanzi J, Mitchell N, M’Soka J, Msuha M, Mweetwa T, Nyahongo J, Rosenblatt E, Schuette P, Sillero-Zubiri C, Sinclair ARE, Stanley Price MR, Zimmermann A, Pettorelli N, Cadotte M (2015). Developing fencing policies for dryland ecosystems. Journal of Applied Ecology.

[ref-13] Dutta T, Sharma S, Maldonado JE, Wood TC, Panwar HS, Seidensticker J (2013a). Gene flow and demographic history of leopards (*Panthera pardus*) in the central Indian highlands. Evolutionary Applications.

[ref-14] Dutta T, Sharma S, Maldonado JE, Wood TC, Panwar HS, Seidensticker J (2013b). Fine-scale population genetic structure in a wide-ranging carnivore, the leopard (*Panthera pardus fusca*) in central India. Diversity and Distributions.

[ref-15] Dutta T, Sharma S, McRae BH, Roy PS, DeFries R (2016). Connecting the dots: mapping habitat connectivity for tigers in central India. Regional Environmental Change.

[ref-16] Farhadiniaa MS, Ahmadi M, Sharbafi E, Khosravi S, Alinezhad H, Macdonald DW (2015). Leveraging trans-boundary conservation partnerships: Persistence of Persian leopard (*Panthera pardus saxicolor*) in the Iranian Caucasus. Biological Conservation.

[ref-17] Fattebert J, Robinson HS, Balme G, Slotow R, Hunter L (2015). Structural habitat predicts functional dispersal habitat of a large carnivore: how leopards change spots. Ecological Applications.

[ref-18] Ghoddousi A, Kh. Hamidi A, Ghadirian T, Ashayeri D, Khorozyan I (2010). The status of the endangered persian leopard *Panthera pardus* saxicolor in Bamu National Park, Iran. Oryx.

[ref-19] Ghoshal A, Bhatnagar YV, Pandav B, Sharma K, Mishra C, Raghunath R, Suryawanshi KR (2019). Assessing changes in distribution of the Endangered snow leopard *Panthera uncia* and its wild prey over 2 decades in the Indian Himalaya through interview-based occupancy surveys. Oryx.

[ref-20] Grassman LIJ (1999). Ecology and behaviour of the Indochinese leopard in Kaeng Krachan National Park, Thailand. Natural History Bulletin of the Siam Society.

[ref-25] Gubbi S, Poornesha HC, Daithota A, Nagashettihalli H (2014). Roads emerging as a critical threat to leopards in India. Cat News.

[ref-24] Gubbi S, Nagashettihalli H, Bhat R, Poornesha HC, Anoop A, Madhusudan MD (2017a). Ecology and conservation of leopards in protected and multiple use forests in Karnataka.

[ref-21] Gubbi S, Harish NS, Kolekar A, Poornesha HC, Reddy V, Mumtaz J, Madhusudan MD (2017b). From intent to action: a case study for the expansion of tiger conservation from southern India. Global Ecology and Conservation.

[ref-22] Gubbi S, Kolekar A, Chakraborty P, Kumara V (2019). Big cat in well: an unconventional threat to leopards in southern India. Oryx.

[ref-23] Gubbi S, Kolekar A, Kumara V (in press). Policy to on-ground action: Evaluating a conflict policy guideline for leopards in India. Journal of International Wildlife Law & Policy.

[ref-26] Harihar A, Pandav B, Goyal SP (2009). Density of leopards (*Panthera pardus*) in the Chilla Range of Rajaji National Park, Uttarakhand, India. Mammalia.

[ref-27] Henschel P, Hunter LTB, Coad L, Abernethy KA, Mühlenberg M (2011). Leopard prey choice in the Congo Basin rainforest suggests exploitative competition with human bushmeat hunters. Journal of Zoology.

[ref-28] Hines JE, Nichols JD, Royle JA, MacKenzie DI, Gopalaswamy AM, Kumar NS, Karanth KU (2010). Tigers on trails: occupancy modeling for cluster sampling. Ecological Applications.

[ref-29] International Union for Conservation of Nature (2016). Keeping leopards in the spot(light) at CITES. https://www.iucn.org/news/species/201610/keeping-leopards-spotlight-cites.

[ref-30] Jacobson AP, Gerngross P, Lemeris JR, Schoonover RF, Anco C, Breitenmoser-Würsten C, Durant SM, Farhadinia MS, Henschel P, Kamler JF, Laguardia A, Rostro-García S, Stein AB, Dollar L (2016). Leopard (*Panthera pardus*) status, distribution, and the research efforts across its range. PeerJ.

[ref-31] Kalle R, Ramesh T, Qureshi Q, Sankar K (2011). Density of tiger and leopard in a tropical deciduous forest of Mudumalai Tiger Reserve, southern India, as estimated using photographic capture–recapture sampling. Acta Theriologica.

[ref-32] Kendall WL, White GC (2009). A cautionary note on substituting spatial subunits for repeated temporal sampling in studies of site occupancy. Journal of Applied Ecology.

[ref-33] Khorozyan I (2003). Habitat preferences by the Persian Leopard (*Panthera pardus saxicolor* Pocock, 1927) in Armenia. Zoology in the Middle East.

[ref-34] Khorozyan IG, Malkhasyan AG, Abramov AV (2008). Presence-absence surveys of prey and their use in predicting leopard (*Panthera pardus*) densities: a case study from Armenia. Integrative Zoology.

[ref-35] Khorozyan IG, Malkhasyan AG, Asmaryan SG, Abramov AV, Cushman SA, Huettmann F (2010). Using geographical mapping and occupancy modeling to study the distribution of the critically endangered leopard (*Panthera pardus*) population in Armenia. Spatial Complexity, Informatics and Wildlife Conservation.

[ref-36] Laguardia A, Kamler JF, Li S, Zhang C, Zhou Z, Shi K (2017). The current distribution and status of leopards *Panthera pardus* in China. Oryx.

[ref-37] Linkie M, Dinata Y, Nugroho A, Haidir IA (2007). Estimating occupancy of a data deficient mammalian species living in tropical rainforests: sun bears in the Kerinci Seblat region, Sumatra. Biological Conservation.

[ref-38] Linkie M, Guillera-Arroita G, Smith J, Rayan DM (2010). Monitoring tigers with confidence. Integrative Zoology.

[ref-39] MacKenzie DI, Nichols JD, Hines JE, Knuston MG, Franklin AB (2003). Estimating site occupancy, colonization, and local extinction when a species is detected imperfectly. Ecology.

[ref-40] MacKenzie DI, Nichols JD, Royle JA, Pollock KH, Hines JE, Bailey LL (2006). Occupancy estimation and modeling: inferring patterns and dynamics of species occurrence.

[ref-69] MacKenzie DI, Bailey LL (2004). Assessing the fit of site-occupancy models. Journal of Agricultural, Biological, and Environmental Statistics.

[ref-67] Mackenzie DI, Royle JA (2005). Designing occupancy studies: General advice and allocating survey effort. Journal of Applied Ecology.

[ref-68] MacKenzie DI, Nichols DJ, Royle A, Pollock KH, Bailey LL, Hines JE (2018). Occupancy in community-level studies. Occupancy Estimation and Modeling: Inferring Patterns and Dynamics of Species Occurrence.

[ref-42] Mondol S, Sridhar V, Yadav P, Gubbi S, Ramakrishnan U (2015). Tracing the geographic origin of traded leopard body parts in the Indian subcontinent with DNA-based assignment tests. Conservation Biology.

[ref-43] Naha D, Sathyakumar S, Rawat GS (2018). Understanding drivers of human-leopard conflicts in the Indian Himalayan region: spatio-temporal patterns of conflicts and perception of local communities towards conserving large carnivores. PLOS ONE.

[ref-44] Norton PM, Henley SR (1987). Home range and movements of male leopards in the Cedarberg Wilderness Area, Cape Province. South African Journal of Wildlife Research.

[ref-45] Nowell K (2014). Review of implementation of Resolution Conf. 12.5 (Rev. CoP16) on Conservation and trade in tigers and other Appendix-I Asian big cats. IUCN/SSC Cat Specialist Group. CITES SC65 Doc.38 Annex 1. http://admin.indiaenvironmentportal.org.in/files/file/Asian%20big%20cat%20species.pdf.

[ref-46] Odden M, Athreya V, Rattan S, Linnell JDC (2014). Adaptable neighbours: movement patterns of GPS-collared leopards in human dominated landscapes in India. PLOS ONE.

[ref-47] Odden M, Wegge P (2005). Spacing and activity patterns of leopards *Panthera pardus* in the Royal Bardia National Park, Nepal. Wildlife Biology.

[ref-48] Pudyatmoko S (2017). Free-ranging livestock influence species richness, occupancy, and daily behaviour of wild mammalian species in Baluran National Park, Indonesia. Mammalian Biology.

[ref-49] Ray JC, Hunter L, Zigouris J (2005). Setting conservation and research priorities for larger African carnivores.

[ref-50] Raza RH, Chauhan DS, Pasha MKS, Sinha S (2012). Illuminating the blind spot: a study on illegal trade in Leopard Parts in India (2001–2010).

[ref-51] Santika T, McAlpine CA, Lunney D, Wilson KA, Rhodes JR, Thuiller W (2014). Modelling species distributional shifts across broad spatial extents by linking dynamic occupancy models with public-based surveys. Diversity and Distributions.

[ref-52] Sasidhran S, Adila N, Hamdan MS, Samantha LD, Aziz N, Kamarudin N, Puan CL, Turner E, Azhar B (2016). Habitat occupancy patterns and activity rate of native mammals in tropical fragmented peat swamp reserves in Peninsular Malaysia. Forest Ecology and Management.

[ref-53] Sharma S, Dutta T, Maldonado JE, Wood TC, Panwar HS, Seidensticker J (2013). Spatial genetic analysis reveals high connectivity of tiger (Panthera tigris) populations in the Satpura-Maikal landscape of Central India. Ecology and Evolution.

[ref-54] Shehzad W, Nawaz MA, Pompanon F, Coissac E, Riaz T, Shah SA, Taberlet P (2015). Forest without prey: livestock sustain a leopard *Panthera pardus* population in Pakistan. Oryx.

[ref-55] Simcharoen S, Barlow ACD, Simcharoen A, Smith JLD (2008). Home range size and daytime habitat selection of leopards in Huai Kha Khaeng Wildlife Sanctuary, Thailand. Biological Conservation.

[ref-56] Stein AB, Athreya V, Gerngross P, Balme G, Henschel P, Karanth U, Miquelle D, Rostro-Garcia S, Kamler JF, Laguardia A, Khorozyan I, Ghoddousi A (2020). *Panthera pardus* (amended version of 2019 assessment). IUCN Red List of Threatened Species.

[ref-57] Stein AB, Hayssen V (2013). *Panthera pardus* (Carnivora: Felidae). Mammalian Species.

[ref-58] Strampelli P, Andresen L, Everatt KT, Somers MJ, Rowcliffe JM (2018). Habitat use responses of the African leopard in a human-disturbed region of rural Mozambique. Mammalian Biology.

[ref-59] Sunarto S, Kelly MJ, Parakkasi K, Klenzendorf S, Septayuda E, Kurniawan H (2012). Tigers need cover: multi-scale occupancy study of the big cat in Sumatran forest and plantation landscapes. PLOS ONE.

[ref-60] Taubmann J, Sharma K, Uulu KZ, Hines JE, Mishra C (2016). Status assessment of the Endangered snow leopard *Panthera uncia* and other large mammals in the Kyrgyz Alay, using community knowledge corrected for imperfect detection. Oryx.

[ref-61] Thapa A, Shah KB, Pokheral CP, Paudel R, Adhikari D, Bhattarai P, Cruz NJ, Aryal A (2017). Combined land cover changes and habitat occupancy to understand corridor status of Laljhadi-Mohana wildlife corridor, Nepal. European Journal of Wildlife Research.

[ref-62] Thatte P, Chandramouli A, Tyagi A, Patel K, Baro P, Chhattani H, Ramakrishnan U, Burridge C (2020). Human footprint differentially impacts genetic connectivity of four wide-ranging mammals in a fragmented landscape. Diversity and Distributions.

[ref-63] Thatte P, Joshi A, Vaidyanatha S, Landguth E, Ramakrishnan U (2018). Maintaining tiger connectivity and minimizing extinction into the next century: insights from landscape genetics and spatially-explicit simulations. Biological Conservation.

[ref-70] Wang T, Feng L, Yang H, Han B, Zhao Y, Juan L, Lü X, Zou L, Li T, Xiao W, Mou P, Smith JLD, Ge J (2017). A science-based approach to guide Amur leopard recovery in China. Biological Conservation.

[ref-64] Wibisono HT, Linkie M, Guillera-Arroita G, Smith JA, Sunarto, Pusparini W, Asriadi, Baroto P, Brickle N, Dinata Y, Gemita E, Gunaryadi D, Haidir IA, Herwansyah, Karina I, Kiswayadi D, Kristiantono D, Kurniawan H, Lahoz-Monfort JJ, Leader-Williams N, Maddox T, Martyr DJ, Maryati, Nugroho A, Parakkasi K, Priatna D, Ramadiyanta E, Ramono WS, Reddy GV, Rood EJJ, Saputra DY, Sarimudi A, Salampessy A, Septayuda E, Suhartono T, Sumantri A, Susilo, Tanjung I, Tarmizi, Yulianto K, Yunus M, Zulfahmi (2011). Population status of a cryptic top predator: an island-wide assessment of tigers in Sumatran rainforests. PLOS ONE.

[ref-65] Williams BK, Nichols JD, Conroy MJ (2002). Analysis and management of animal populations.

[ref-66] Witmer GW (2005). Wildlife population monitoring: some practical considerations. Wildlife Research.

